# Synergistic Reducing Effect for Synthesis of Well-Defined Au Nanooctopods With Ultra-Narrow Plasmon Band Width and High Photothermal Conversion Efficiency

**DOI:** 10.3389/fchem.2018.00335

**Published:** 2018-08-10

**Authors:** Yi-Xin Chang, Hui-Min Gao, Ning-Ning Zhang, Xing-Fu Tao, Tianmeng Sun, Junhu Zhang, Zhong-Yuan Lu, Kun Liu, Bai Yang

**Affiliations:** ^1^State Key Laboratory of Supramolecular Structure and Materials, College of Chemistry, Jilin University, Changchun, China; ^2^Laboratory of Theoretical and Computational Chemistry, Institute of Theoretical Chemistry, Jilin University, Changchun, China; ^3^The First Bethune Hospital and Institute of Immunology, Jilin University, Changchun, China; ^4^State Key Laboratory of Applied Optics, Changchun Institute of Optics, Fine Mechanics and Physics, Chinese Academy of Sciences, Changchun, China

**Keywords:** nanooctopods, nanobranch, nanostar, ultra-narrow plasmon band width, photothermal conversion efficiency, photoacoustic imaging, synergistic reducing effect

## Abstract

Branched Au nanoparticles have attracted intense interest owing to their remarkable properties and a wide variety of potential applications in surface-enhanced Raman spectroscopy (SERS), photothermal therapy, photoacoustic imaging, and biomedicines. The morphology and spatial arrangement of branches play the most crucial role in the determination of their properties and applications. However, it is still a synthetic challenge to control the exact arm numbers of branches with specific spatial arrangements. Here we report a facile method for the kinetically controlled growth of Au nanooctopods (NOPs) with a high yield (81%), monodispersity, and reproducibility by using the synergistic reducing effect of ascorbic acid and 1-methylpyrrolidine. The NOPs have eight arms elongated along <111> directions with uniform arm lengths. Due to their well-defined size and shape, NOPs show ultra-narrow surface plasmon band width with a full width at half maximum of only 76 nm (0.20 eV). Upon irradiation with laser, the NOPs possessed excellent photothermal conversion efficiencies up to 83.0% and photoacoustic imaging properties. This work highlights the future prospects of using NOPs with desired physicochemical properties for biomedical applications.

## Introduction

Research on Au nanoparticles has been boosted by diverse applications because of their unique size- and shape-dependent properties (Link and El-Sayed, 2000; Kelly et al., 2003; Li et al., 2014; Boles et al., 2016). The past decade has witnessed spectacular success in developing a myriad of methods for shape-controlled synthesis of anisotropic Au nanoparticles with well-defined structures, such as triangles (Malikova et al., 2002), prisms (Shankar et al., 2004), rods (Jana et al., 2001; Nikoobakht and El-Sayed, 2003), cubes (Sun and Xia, 2002; Kim et al., 2004; Zhang, J. et al., 2010), shells (Averitt et al., 1997), stars (Hao et al., 2004; Nehl et al., 2006; Kumar et al., 2007), bipyramids (Liu and Guyot-Sionnest, 2005), cages (Skrabalak et al., 2008), etc. Among them, Au nanobranches with a center core and several protruding arms with sharp tips have drawn enormous attention due to their excellent localized surface plasmon resonance (LSPR) properties (Chen et al., 2003; Guerrero-Martínez et al., 2011; Lim and Xia, 2011; Ye et al., 2015). The arm-length dependent LSPR band in the biological window (650–1,350 nm), remarkable enhanced local E-field at their sharp tips, and relative high cellular uptake and low cytotoxicity make Au nanobranches very attractive for bio-applications, such as SERS-based sensing (Khoury and Vo-Dinh, 2008; Indrasekara et al., 2014), photoacoustic imaging (Wei et al., 2009; Cheng et al., 2014), photothermal therapy (Yuan et al., 2012; Wang et al., 2013), and nanomedicines (Dam et al., 2012), etc.

Many of these applications rely on single wavelength laser to excite the plasmon resonance. To maximize the radiation efficiency and minimize the toxicity and negative side effect in bio-applications, Au nanobranches with narrow plasmon band matching with the laser wavelength is strongly desired. In addition, a narrower plasmon peak width implies a smaller plasmon damping and a larger local electric field enhancement of the nanoparticles (Sönnichsen et al., 2002). Narrow plasmon linewidth of nanoparticles is vital in many plasmonic applications (Zhu et al., 2016), such as monitoring their electron transfer to other materials (Hoggard et al., 2013), studying chemical bonding on their surface (Zijlstra et al., 2012), and developing ultrasensitive chemical and biological LSPR sensors (Mayer and Hafner, 2011). Generally, the extinction spectra of nanobranches, however, exhibit very broad UV-Vis-NIR peaks (inhomogeneous broadening effects) due to unavoidable shape differences of individual Au nanobranches, that is, the number, size, and orientation of their arms vary greatly among them. Although Au nanooctopods can be synthesized with the cubic Au seeds from previous literature protocols, the arms of the octopods were too short to exhibit highly branched architectures and sharp tips. In addition, the Au nanooctopods showed broad band due to their size and structural inhomogeneity (Smith et al., 2016). Therefore, the lack of reliable synthetic methods for highly branched Au nanoparticles with accurate control of the number of arms, spatial arrangement of arms, arm size, high yield as well as reproducibility hinders the fundamental understanding of their properties and the assessment of their potentials (Niu et al., 2015).

Herein, we report a facile kinetically controlled growth of monodisperse NOPs with a high yield and narrow LSPR band by using 1-methylpyrrolidine (1-MP) and ascorbic acid (AA) as effective co-reducing agents for HAuCl_4_ at room temperature. With the presence of single-crystalline Au seeds, the high reduction rate of Au precursor leads to the growth of eight arms along <111> directions of the seeds. The NOPs show strong and ultra-narrow LSPR peak in the range of 660–720 nm. They exhibit remarkable photothermal conversion efficiencies up to 83.0% and a photoacoustic response upon 680 nm laser irradiation. These NOPs hold great promise of highly efficient photothermal conversion and photoacoustic dual-functional agents.

## Materials and methods

### Materials

Hexadecyltrimethylammonium bromide (CTAB), 1-methylpyrrolidine (1-MP), gold (III) chloride trihydrate (HAuCl_4_·3H_2_O), ascorbic acid (AA), and sodium borohydride (NaBH_4_) were purchased from Sigma-Aldrich. Monomethoxy-poly (ethylene glycol)-thiol (mPEG-SH, molecular weight = 5,000 g/mol) was purchased from JenKem Technology. All the reagents were used as received without further purification. Deionized water (18.2 MΩ·cm) was used for all solution preparations.

### Synthesis of Au seeds

The gold seed nanoparticles were prepared according to the method reported previously with a slight modification (Nikoobakht and El-Sayed, 2003). CTAB solution (3.5 mL, 0.14 M) was mixed with of HAuCl_4_ (0.125 mL, 15 mM). A freshly prepared, ice-cold NaBH_4_ solution (0.50 mL, 0.010 M) was injected to the mixture, resulting in the formation of a brownish yellow solution. The seed solution was vigorously stirred for 120 s and then kept at 25°C.

### Synthesis of Au NOPs

The aqueous solutions of HAuCl_4_ (15 mM, 0.38 mL), CTAB (0.40 M, 2.375 mL), and 1-MP (1.0 M, 0.90 mL) were added in a glass vial, named solution A. Deionized water (6.12 mL), AA (0.10 M, 225 μL), and seeds solution (1.8 μL) were added in another glass vial, named solution B. Solution B was quickly added to solution A, and the reaction mixture was incubated at 25°C for 5 min.

### PEGylation of Au NOPs

The as-synthesized NOPs (1.0 mL) were purified by centrifugation at 9,000 rpm for 15 min. The precipitation was redispersed by 1.0 mL aqueous solution of mPEG-SH (0.10 mg/mL). The mixture was ultra-sonicated for 5 min and stirred over night at room temperature.

### Materials characterization

The size, morphology, and structure of NOPs were studied by using JEOL JEM-2100F and Hitachi H800 transmission electron microscope (TEM) with an accelerating voltage of 200 and 175 kV, respectively. The UV-Vis-NIR extinction spectra were recorded using a Lambda 950 (PerkinElmer) Spectrometer. The purification of NOPs was performed by using Eppendorf Centrifuge 5430R at 25°C. X-Ray diffraction (XRD) measurements of NOPs solutions drop-coated onto glass substrates were done on Empyrean (PANalytical B. V.) operating at a voltage of 40 kV.

### Simulation of the capping effect of 1-MP

Density Functional Theory calculations were performed by means of the Vienna Ab Initio Simulation Package (VASP) (Hafner, 2008) using the Perdew-Burke-Ernzerhof (PBE) generalized-gradient approximation (Perdew et al., 1996). The interactions between valence electrons and ion cores were treated by Blöchl's all-electron-like projector augmented wave (PAW) method (Blöchl, 1994). The plane-wave cutoff for the wave functions was 400 eV throughout. For all calculations, we used a (4 × 4) supercell consisting of a four-layer slab with vacuum thickness of about 20 Å and molecules adsorbed on one side of the slab. The adsorbed molecule and the top three layers of Au were allowed to relax. The Brillouin zone integration was performed using a cell size dependent Monkhorst–Pack *k* point sampling (Monkhorst and Pack, 1976) and the *k*-point mesh was 3 × 3 × 1 for surface reconstruction and 7 × 7 × 1 for single-molecule adsorption. Ionic relaxation for all stable structures was carried out until all forces were smaller than 0.02 eV/Å. The adsorption energy for molecules on Au was calculated as follows:

$$\begin{array}{l} \Delta E_{ads}\;=\;E_{molecule/Au}-E_{molecule}-E_{Au} \end{array}$$

where *E*_molecule/Au_ is the total energy of the Au surface together with the adsorbed molecule, *E*_molecule_ is the total energy of the free molecule, and *E*_Au_ is the total energy of the bare Au surface. With this method, the negative values of adsorption energy suggest that the adsorption configuration is more stable than the corresponding bare Au surface and the free molecule.

### Laser-induced heat conversion of Au NOPs

A diode laser at 660 nm (LEO photonics Co. Ltd.) was employed in the experiments. PEG-modified NOPs (1.0 mL) in a quartz cuvette (1-cm path length) was irradiated by the laser with tunable power densities (0.50–2.0 W/cm^2^). PEG-modified NOPs with different optical densities (ODs) (0.10–1.0) were irradiated by the laser at 1.0 W/cm^2^. Deionized water was used as a negative control. The laser spot was adjusted to cover the entire surface of the sample. Real-time thermal imaging and the maximum temperature was recorded by a FLIR A310 infrared camera.

### *In vitro* photoacoustic imaging of Au NOPs

PEG-modified NOPs solutions with different optical densities were introduced into agar-gel cylinders and put in deionized water at a consistent depth. Photoacoustic images were obtained at 680 nm wavelength by MSOT inVision 128 small animal scanner.

## Results and discussion

**Figure 1:**
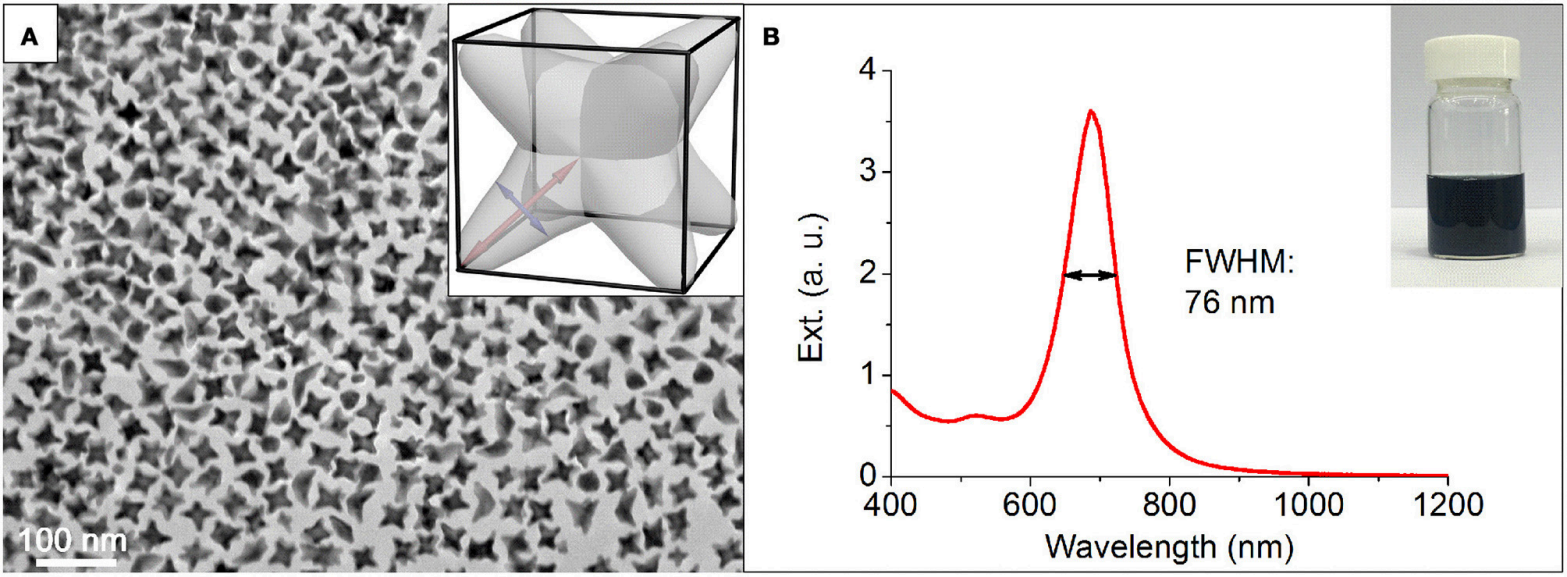
**(A)** Representative TEM image and **(B)** extinction spectra of NOPs. Inset in **(A)**: scheme of an individual NOP. Red and blue lines represent the arm length and width, respectively. Inset in **(B)**: optical photograph of the NOP solution.

The NOPs were prepared by the regrowth from well-defined single-crystalline Au seeds (see section Materials and Methods for details). In a typical synthesis, a seed solution of Au seeds and ascorbic acid (AA) were quickly added into a fresh-prepared growth solution containing HAuCl_4_ and 1-methylpyrrolidine (1-MP) in the presence of hexadecyltrimethylammonium bromide (CTAB) at room temperature and final pH of 11.3. Both AA and 1-MP (Newman and Blanchard, 2006) were reducing agents for HAuCl_4_, and the basic pH increased the reduction rates (vide infra). CTAB was used as a surface capping agent to provide the colloidal stability for the synthesized nanoparticles. After the mixing, the color of the solution immediately turned from colorless to peacock blue in about 5 min, indicating the formation of NOPs. Transmission electron microscopy (TEM) study (Figure 1A) of NOPs reveals the NOPs with a high yield (81%) of possessed eight arms with a uniform arm length and width of 19.5 ± 1.4 and 9.4 ± 1.0 nm, respectively. The NOPs also showed an extraordinary LSPR peak at 680 nm with a FWHM (Full width at half maximum) as narrow as 76 nm (0.20 eV) (Mets et al., 2012), which demonstrates high monodispersity of the NOPs (Figure 1B).

**Figure 2:**
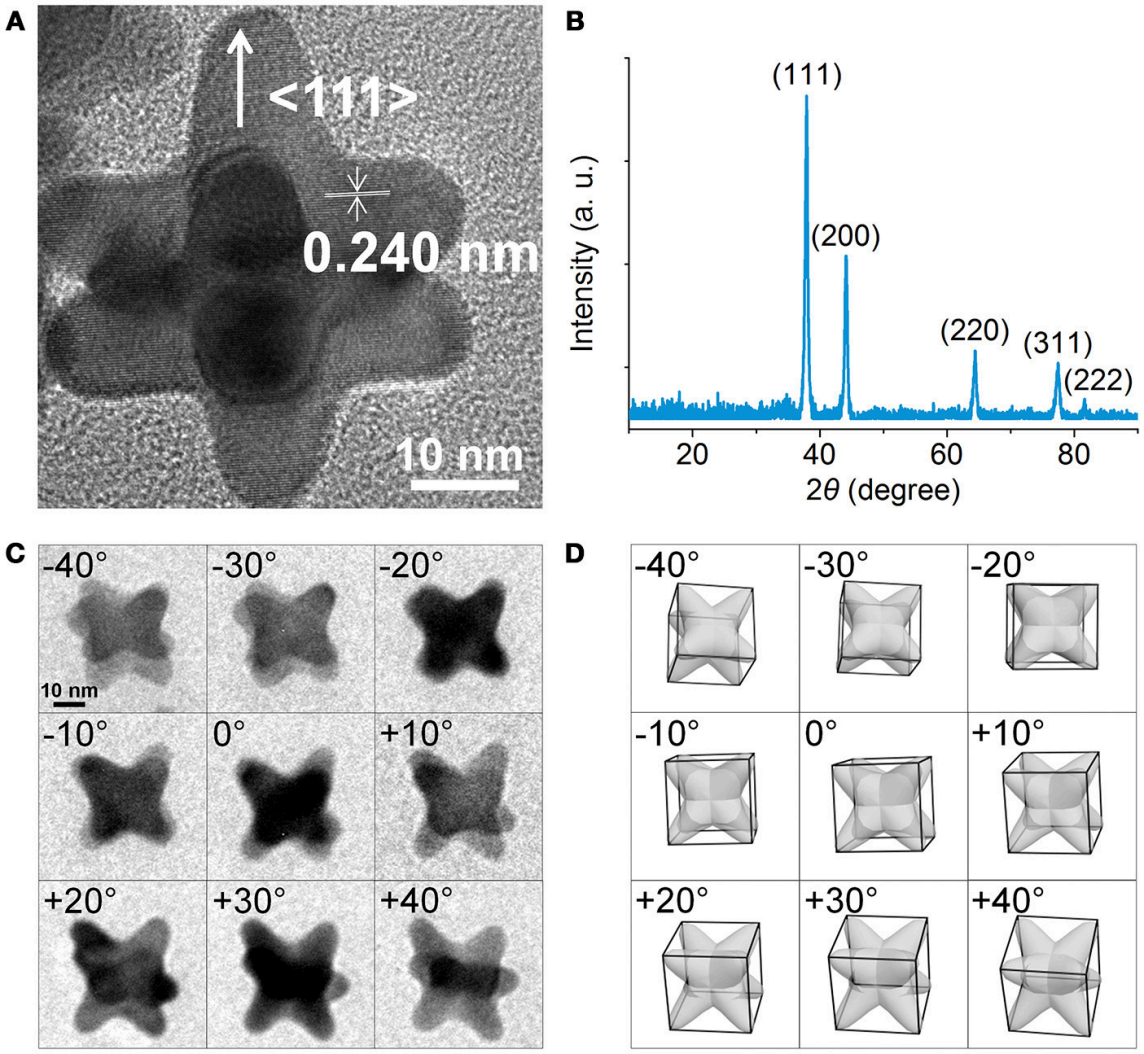
**(A)** HRTEM image of an individual NOP. **(B)** Powder X-ray Diffraction of the NOPs. **(C)** 3D-TEM images of NOPs tilted from ca. −40° to +40°. **(D)** Corresponding 3D model images with same views from ca. −40° to +40°.

Figure 2A shows a high-resolution TEM image of an individual NOP, where periodic lattice fringes can be clearly resolved. The distance between adjacent lattice fringes was about 0.240 nm, corresponding to the d-spacing of Au {111} planes. The X-ray diffraction (XRD) pattern recorded on a glass substrate was displayed in Figure 2B. The diffraction peaks were assigned to (111), (200), (220), (311), (222) planes of face-centered cubic Au, respectively (JCPDS No. 04-0784). It is worth noting that although the ratio between the intensities of the (200) to (111) diffraction peaks was similar to the conventional values (0.50 vs. 0.52), the ratios of (220)/(111) and (220)/(200) were significantly lower than the conventional values (0.21 vs. 0.32) and (0.42 vs. 0.62), respectively. This result indicates that the NOPs were abundant in (111) and (200) facets. Close inspection and analysis of the image (Figure 2A) reveals that the arms grew from the eight corners of Au seeds along <111> directions to form octopod structure. To further understand the spatial arrangement of the arms, three dimensional (3D)-TEM images taken from various tilting angles were consistent with our model of NOPs at different views (Figures 2C,D).

The formation of anisotropic structure requires breaking the face-centered cubic symmetry of Au crystals. Therefore, the growth mechanism is important for understanding how the arms arise from Au seeds. In general, thermodynamically controlled nanocrystals with the global minimum in Gibbs free energy can be synthesized by capping agents which effectively reduce the surface free energy of the nanocrystals. To help elucidate this process, we first considered the effect of CTAB and 1-MP as capping agents. Density Functional Theory (DFT) calculations of the adsorption energy of 1-MP on different Au crystal facets indicate that 1-MP taking a flat conformation with the N atom preferentially adsorbed on the top-position of Au atoms (Figure S1). The sequence of 1-MP adsorption energies (Δ*E*_ads_) on different crystallographic facets is in agreement with that of CTAB (Liu et al., 2011), i.e., |Δ*E*_ads_ (111)| < |Δ*E*_ads_ (100)| < |Δ*E*_ads_ (110)|. This result suggests that the NOPs covered by both 1-MP and CTAB would thermodynamically enclosed by higher proportion of {110} facets on the surface, which is conflicting with our analysis of XRD result (Figure 2B).

**Figure 3:**
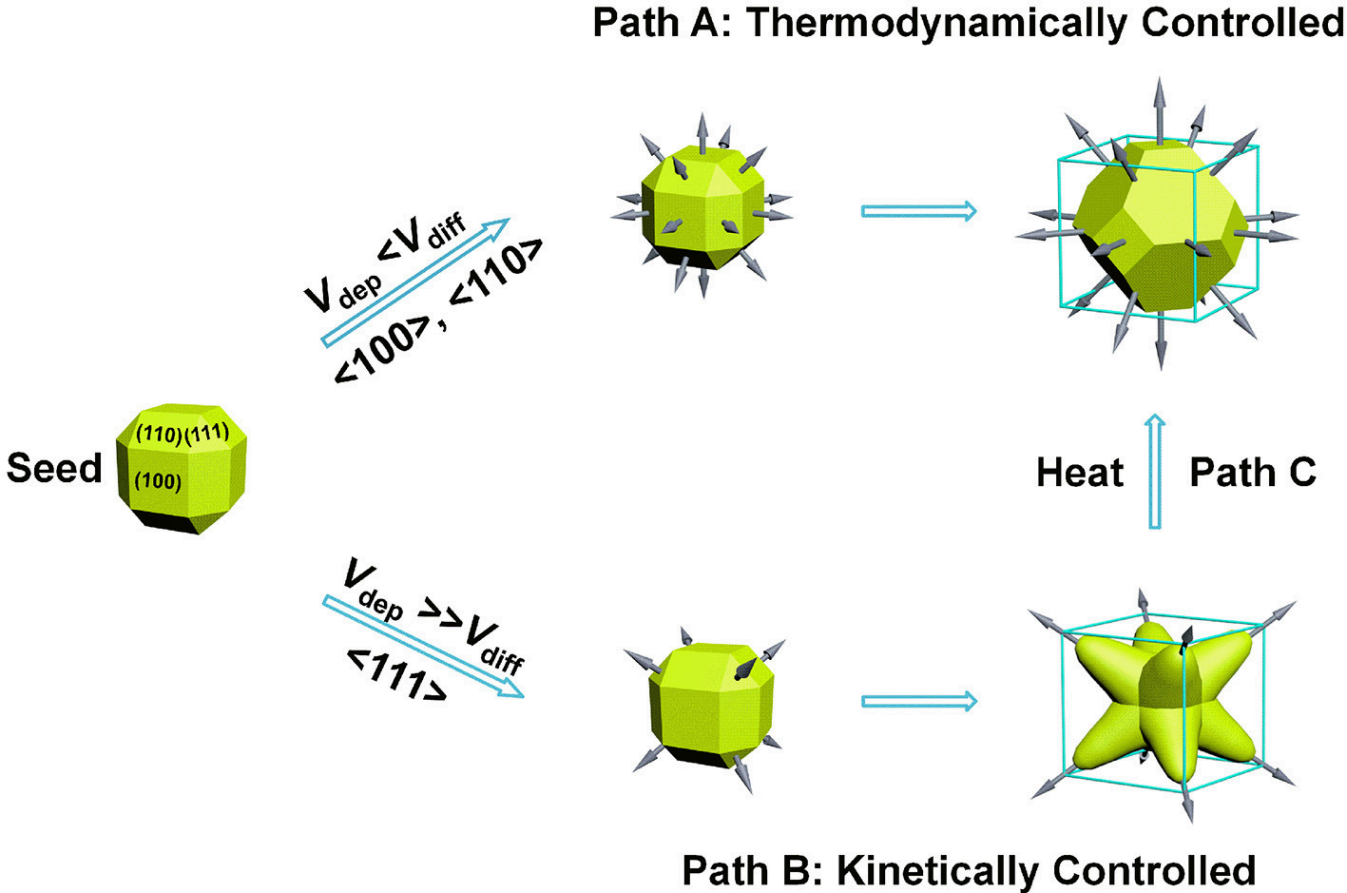
Growth mechanism of kinetically controlled synthesis of NOPs.

Our success in the synthesis of NOPs essentially relied on the kinetic control of growth process (Zhang, H. et al., 2010; Xia et al., 2013, 2015; Wang et al., 2015a). As proposed previously, for seed-mediated growth method, the ratio between the deposition rate (*V*_dep_) and the diffusion rate (*V*_diff_) determines the growth pathway from a single-crystal seed and thereby the morphology of the produced nanocrystals. *V*_dep_ is the rate for the atoms added to the active nanocrystal facets with a higher surface free energy, and *V*_diff_ is the rate for the adatoms migrating to the nanocrystal facets with a lower surface free energy. For a near rhombicuboctahedron-shaped single-crystal seed, due to the preferential binding of CTAB capping agent on the {100} and {110} facets, {111} corner facets are the most active sites as a result of less coverage by the capping agent (Park et al., 2013). For a slow reduction rate, *V*_dep_ ≪ *V*_diff_, most of the adatoms at the corners will migrate to edges and side facets, and the growth will prevail along the <100> and <110> directions, leading to the formation of Wulff shape (Xia et al., 2015), as favored by thermodynamics (Figure 3, Path A). On the contrary, for a fast reduction rate, *V*_dep_ ≫ *V*_diff_, surface diffusion can be ignored and the growth will be switched to the <111> directions, promoting the formation of a kinetically favored octopods as the product (Figure 3, Path B).

Both of *V*_dep_ and *V*_diff_ are kinetic parameters and can be manipulated by varying the ratio between Au seeds and HAuCl_4_, and the rate of reduction of HAuCl_4_. We studied the reductive ability of AA and 1-MP in the absence of Au seeds by monitoring the peak at 395 nm which is attributed from the [CTA]^+^ [AuBr_4_]^−^ complex (Kundu, 2013). It is well-known that AA can reduce HAuCl_4_ to Au^+^ under neutral or acidic condition, but to Au^0^ under strong alkaline condition (Goia and Matijević, 1999). In the present study when only AA was added into the solution A containing [CTA]^+^ [AuBr_4_]^−^ complex at the pH of 11.4, Au nanoparticles with a LSPR peak around 530 nm were formed in 1 min. At the meantime, the peak at 395 nm was remained (Figure S2A), indicating that under strong alkaline condition, AA could not quickly reduce Au^3+^ to Au^+^ as it does under neutral or acid condition. The [CTA]^+^ [AuBr_4_]^−^ complex was completely consumed after 3 h, accompanying by the formation of more Au nanoparticles with quasi-spheres (Figure S3A). This result suggests under strong alkaline condition, although AA can reduce Au^3+^ to form Au nanoparticles, the reduction rate of Au^3+^ to Au^0^ is slow.

On the other side, when only 1-MP was used as reducing agent under the same condition, the peak of [CTA]^+^ [AuBr_4_]^−^ complex was completely vanished in 1 min, while no Au nanoparticles were formed as indicated from the UV-Vis-NIR spectra (Figure S2B). The formation of Au nanoparticles was much slower compared to that of AA. This result reveals that 1-MP is a reducing agent which can reduce Au^3+^ to Au^+^ with a much higher rate compared to AA under basic conditions, but its ability to further reduce Au^+^ to Au^0^ is weaker than that of AA under strong alkaline condition. From the above results we can conclude that if only AA or 1-MP is used as the solo reducing agent, the reduction rate of formation of Au^0^ is not high enough to achieve *V*_dep_ ≫ *V*_diff_, and only spherical or irregular shaped nanoparticles were obtained, respectively (Figure S3). In contrast, when both AA and 1-MP were used, 1-MP can quickly reduce Au^3+^ to Au^+^, which can be further reduced to Au^0^ by AA to reach a high *V*_dep_. Therefore, we conclude that under the strong alkaline condition, AA and 1-MP have a synergistic reducing effect for achieving the condition of *V*_dep_≫*V*_diff_, leading to the formation of NOPs with branches along <111> directions. This result is consistent with the theoretical prediction for the scenario of *V*_dep_ ≫ *V*_diff_ mentioned previously (Xia et al., 2015).

**Figure 4:**
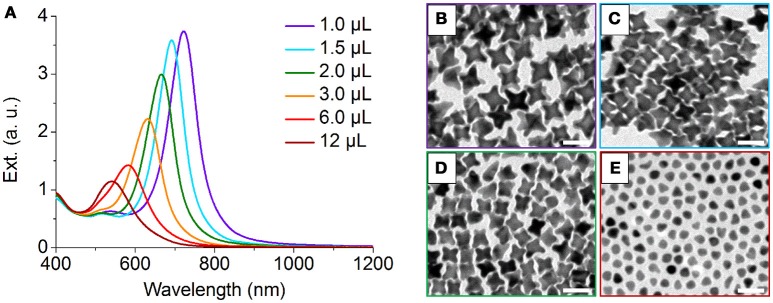
**(A)** UV-Vis-NIR spectra of NOPs by increasing the volume of Au seeds solution from 1.0 to 12 μL added to the growth solution (total volume of 10 mL). **(B–E)** TEM images of NOPs with 1.0, 1.5, 2.0, and 12 μL Au seeds solution added to the growth solution, respectively. Scale bars: 50 nm.

We also performed control experiments to investigate the effect of seed/precursor ratio on the morphology of NOPs. By increasing the volume of Au seeds solution from 1.0 to 3.0 μL with other conditions unchanged, both of the average aspect ratios and lengths of the arms of NOPs can be continuously adjusted from 2.00 to 1.25 and 22.8 to 15.8 nm, respectively (Figure 4, Table S1), that is, the more Au seeds the smaller arms. Further increasing the amount of Au seeds to 12 μL led to only thermodynamically stable product, i.e., quasi-spheres (Figure 4E). This result reveals that when the number of Au atoms available for every seed is smaller, the ratio of *V*_dep_/*V*_diff_ was reduced, leading to thermodynamic products. In addition, this result also demonstrates that one can control the aspect ratio and arm length, as well as LSPR position, by tuning the seed/HAuCl_4_ ratio. It is worth mentioning that the arm length and aspect ratio of NOPs depend on their LSPR position, and the LSPR wavelength was linearly correlated with the aspect ratio of NOPs (*R*^2^ = 0.969) (Figure S4).

Additionally, the reaction temperature also plays a paramount role in determining whether thermodynamic or kinetic control is dominant. High temperatures normally lead to generate thermodynamically controlled nanocrystals. After incubated at higher temperature (60°C) for 4 h, the morphology of as-synthesized NOPs was quickly transformed to Wulff shape with lower surface free energy (Figure S5), which is corresponding to the path C in Figure 3. This result confirms the NOPs are kinetically controlled product, of which the shape changes to thermodynamically stable at high temperatures.

It's worth pointing out that the NOPs are thermodynamically unstable due to a relatively high surface energy at the sharp tips, resulting in changes of their morphology to less anisotropic structures and the blue-shifting of their LSPR bands (Figure S6; Wang et al., 2015b). Therefore, the practical applications of the NOPs are severely limited. In search of the appropriate methods for stabilizing the NOPs, we have tried halogen ions, surfactants (i.e., CTAB), and six thiol compounds, including cysteine, 3-mercaptopropionic acid, glutathione, 11-mercaptopropionic acid, polyetherimide, mPEG-SH. Among them, we found that mPEG-SH modified NOPs showed negligible shifting of their LSPR band in 5 days at room temperature (Figure S7) and good thermal-stability at high temperature (at 75°C for 30 min) (Figure S8).

**Figure 5:**
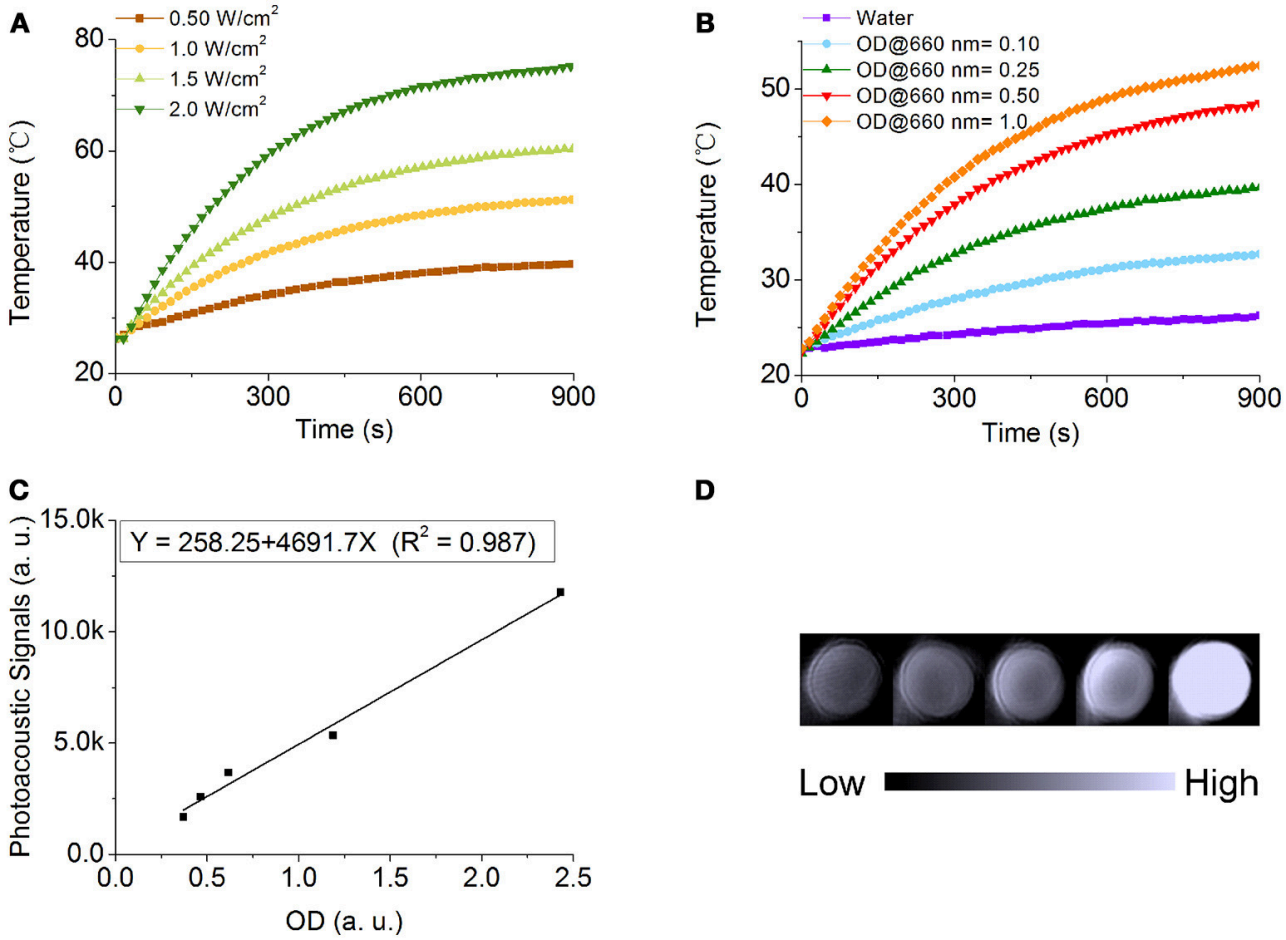
Photothermal conversion and photoacoustic properties of NOPs. **(A)** Temperature variation of NOPs with the optical density at 660 nm (OD_660nm_) of 1.0 irradiated at different laser power densities: 0.50 (brown), 1.0 (yellow), 1.5 (light green), and 2.0 W/cm^2^ (dark green). **(B)** Temperature variation of NOPs with different OD_660nm_ values: 0.10 (blue), 0.25 (green), 0.50 (red), and 1.0 (orange) irradiated at a laser power density of 1.0 W/cm^2^. **(C)** Photoacoustic signals of NOPs as a function of OD_680nm_. **(D)** Photoacoustic images of NOPs.

Owing to the strong LSPR band at 680 nm of NOPs, it is worthy to evaluate their dual potential as photothermal conversion and photoacoustic contrast agents. To evaluate their photothermal conversion performance, aqueous solutions of PEG-modified NOPs with the same optical density at 660 nm (OD_660nm_) of 1.0 were exposed to the 660 nm laser at different power densities (0.50–2.0 W/cm^2^) as shown in Figure 5A. Additionally, PEG-modified NOPs with different optical densities (ODs) (0.10–1.0) were irradiated by the laser at 1.0 W/cm^2^ (Figure 5B). After the laser irradiation, PEG-modified NOPs showed negligible shifting of their LSPR band and kept their morphology unchanged as well (Figure S9). The temperature variation was strongly depended on the laser power and concentration of NOPs (Figures 5A,B). Table S2 lists the photothermal conversion values of NOPs calculated by using a modified model similar to the report by Roper et al. (Roper et al., 2007; Shao et al., 2016). NOPs showed excellent photothermal conversion efficiencies with a maximum value up to 83.0%. The ultrahigh conversion value is attributed to the narrow LSPR band of NOPs. In addition, the solution of NOPs also presented a strong photoacoustic signal at 680 nm wavelength. The photoacoustic intensity was linearly proportional to the concentration of NOPs with *R*^2^ = 0.987, suggesting the NOPs as a promise photoacoustic contrast agent.

## Conclusion

Monodisperse NOPs were synthesized through a kinetically controlled growth with a high yield and ultra-narrow LSPR band width. The synergistic reducing effects of AA and 1-MP were important for the kinetically controlled scenario of *V*_dep_ ≫ *V*_diff_, resulting the formation of the highly branched NOPs along eight <111> directions. The uniform of the number, size, and orientation of their branches makes NOPs their remarkable LSPR properties with ultra-narrow LSPR band. The NOPs offer outstanding properties in bio-applications such as photothermal therapy with high photothermal conversion efficiencies up to 83.0% and a promise photoacoustic contrast agent in photoacoustic imaging. Owing to their excellent LSPR properties, the NOPs are highly promising in diverse applications, such as sensing, nanodevices, catalysis, and bio-applications.

## Author contributions

Y-XC performed the experiments and analyzed the data; H-MG and Z-YL performed the Density Functional Theory calculations; N-NZ and X-FT provided assistance in the experiments; KL, BY, JZ, and TS designed the experiments; KL and Y-XC wrote the manuscript. All authors read and approved the manuscript.

### Conflict of interest statement

The authors declare that the research was conducted in the absence of any commercial or financial relationships that could be construed as a potential conflict of interest.
