# Supplementary material for: Synergistic Reducing Effect for Synthesis of Well-Defined Au Nanooctopods With Ultra-Narrow Plasmon Band Width and High Photothermal Conversion Efficiency
Source: Front Chem. 2018 Aug 10;6:335. doi: 10.3389/fchem.2018.00335 (PMC6096650; doi:10.3389/fchem.2018.00335)
Supplement: Supplementary file 1 [file Data_Sheet_1.pdf]

Supplementary Material

Synergistic Reducing Effect Synthesis of Well-Defined Au Nanooctopods with Ultra-Narrow Plasmon Band Width and High Photothermal Conversion Efficiency

Yi-Xin Chang[1], Hui-Min Gao[2], Ning-Ning Zhang[1], Xing-Fu Tao[1], Tianmeng Sun[3], Junhu Zhang[1,4], Zhong-Yuan Lu[1,2], Kun Liu[1,4*] and Bai Yang[1,4]

[1]State Key Laboratory of Supramolecular Structure and Materials, College of Chemistry, Jilin University, Changchun, China

[2]Laboratory of Theoretical and Computational Chemistry, Institute of Theoretical Chemistry, Jilin University, Changchun, China

[3]The First Bethune Hospital and Institute of Immunology, Jilin University, Changchun, China

[4]State Key Laboratory of Applied Optics, Changchun Institute of Optics, Fine Mechanics and Physics, Chinese Academy of Sciences, Changchun, China

*** Correspondence:** Kun Liu: kliu@jlu.edu.cn

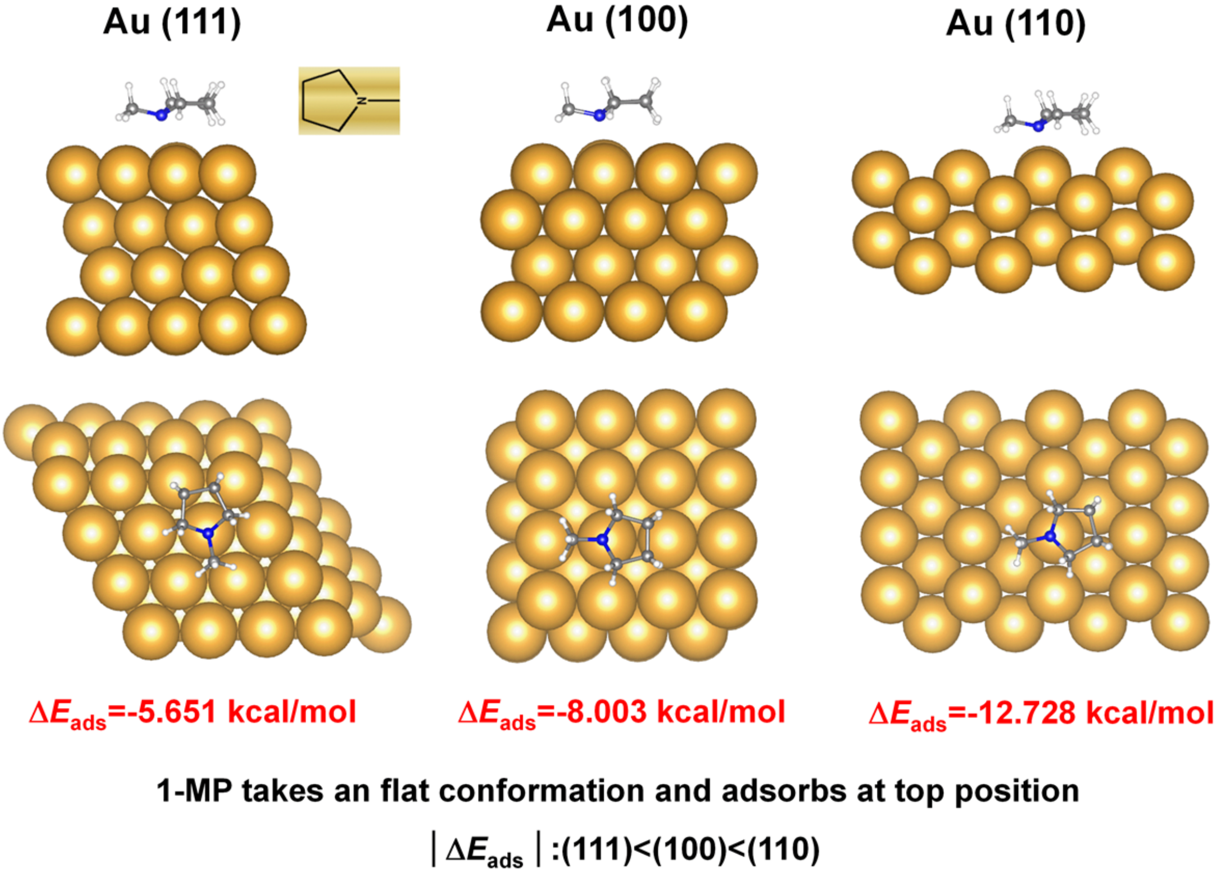

Figure S1. Density Functional Theory (DFT) calculations of the adsorption energy of 1-MP on different Au crystal facets indicated that 1-MP taking an flat conformation with the N atom adsorbed on the top-position of Au atoms. The sequence of 1-MP adsorption energies on different crystallographic facets is $|\Delta E_{ads}\ (111)| < |\Delta E_{ads}\ (100)| < |\Delta E_{ads}\ (110)|$.

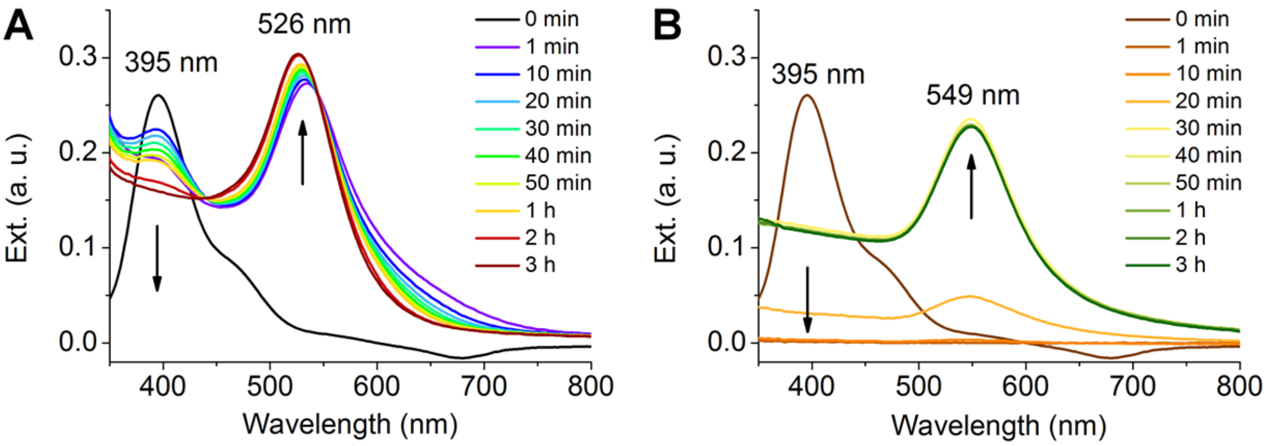

Figure S2. UV-Vis-NIR spectra of (A) AA (2.25 mmol/L) and (B) 1-MP (0.090 mol/L) reacted with $HAuCl_4$ (0.57 mmol/L) at pH = 11.4.

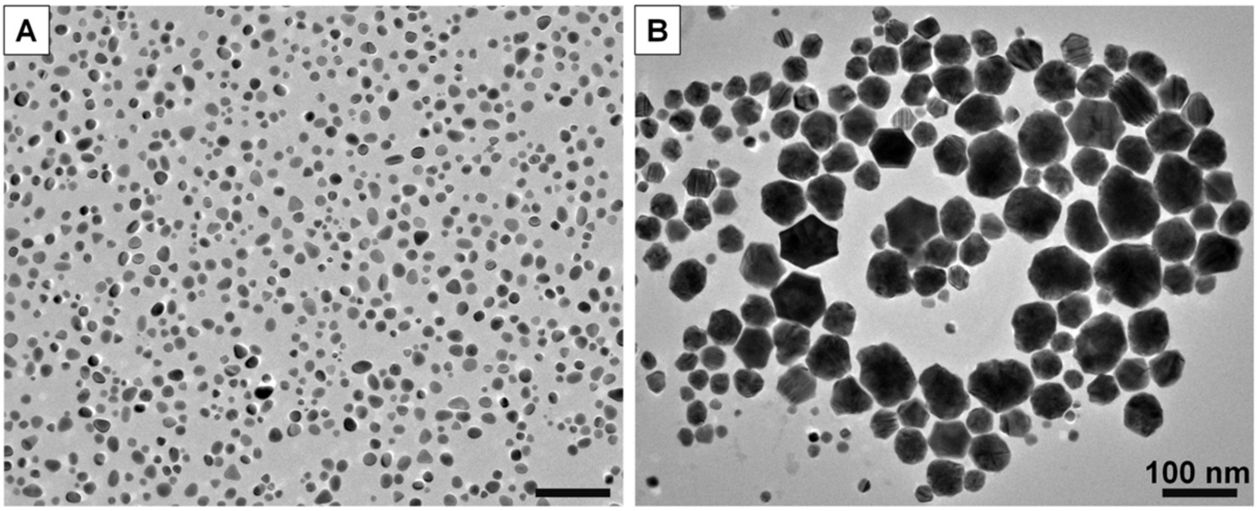

Figure S3. TEM images of Au nanoparticles obtained by only (A) AA (2.25 mmol/L) or (B) 1-MP (0.090 mol/L) as the reducing agent for $HAuCl_4$ (0.57 mmol/L) at pH = 11.4.

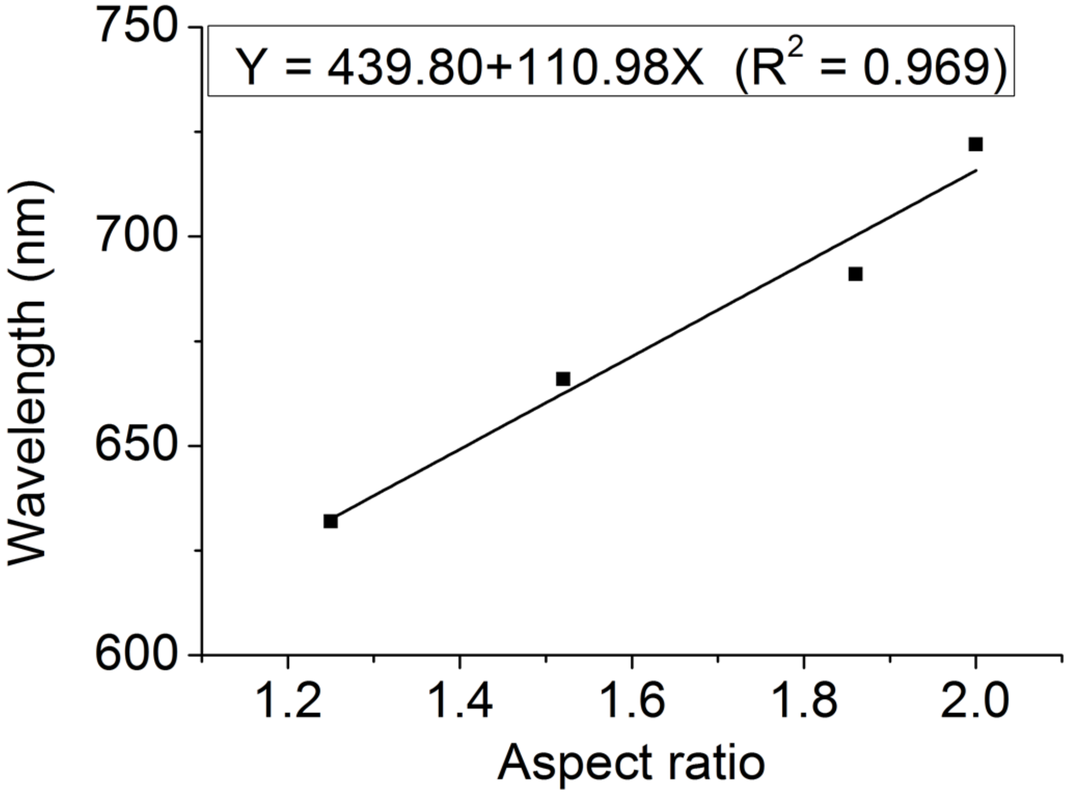

Figure S4. The correlation between the LSPR position and the aspect ratio of NOPs (R^2 = 0.969).

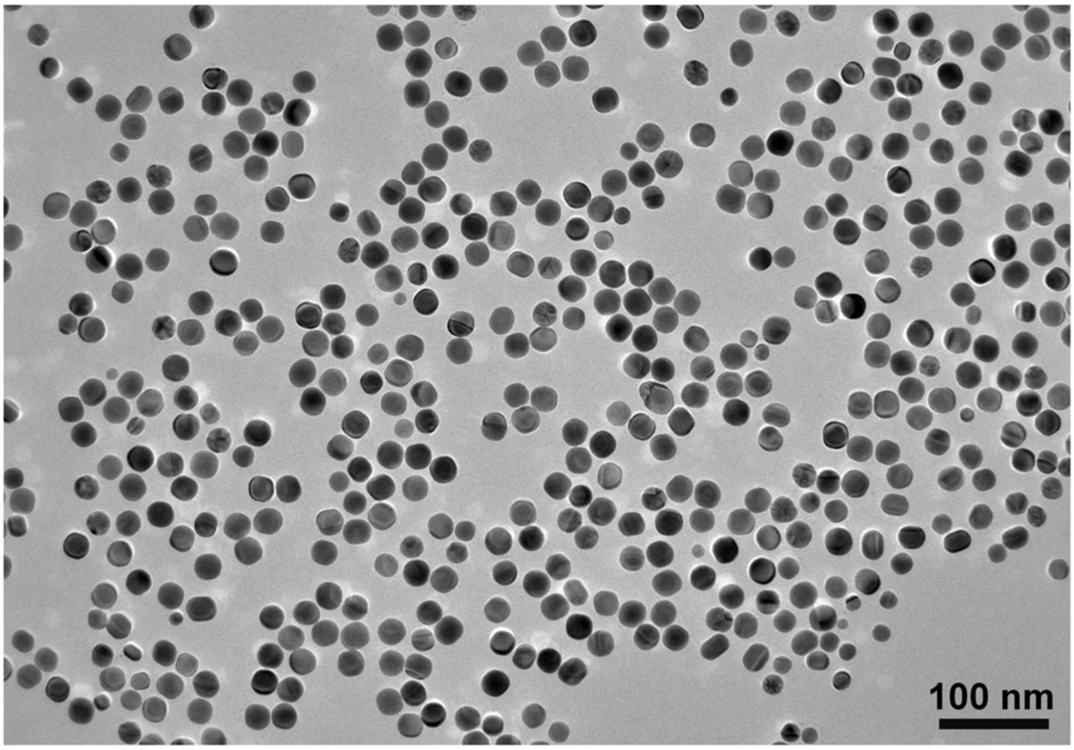

Figure S5. TEM image of NOPs changed to rounded shape after incubated at 60 °C for 4 hours.

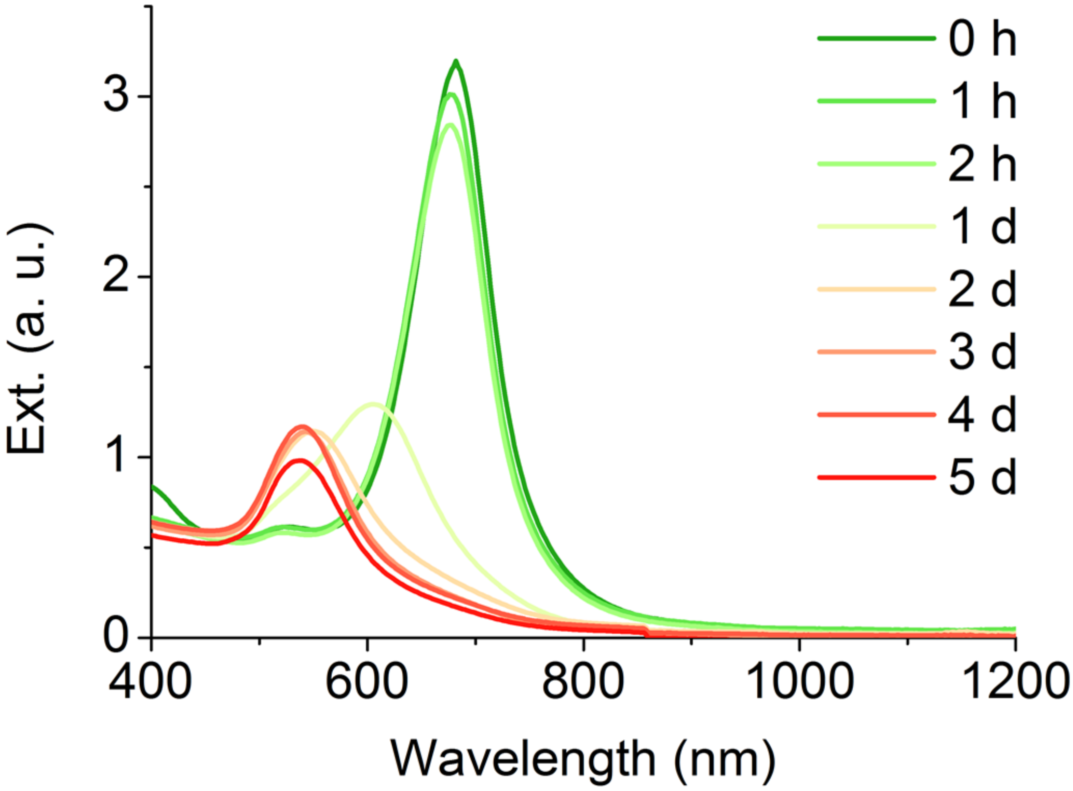

Figure S6. UV-Vis-NIR spectra of NOPs aged at different times indicated in the legends. The blue-shifting of LSPR wavelength indicated the structure change of NOPs to more rounded shapes.

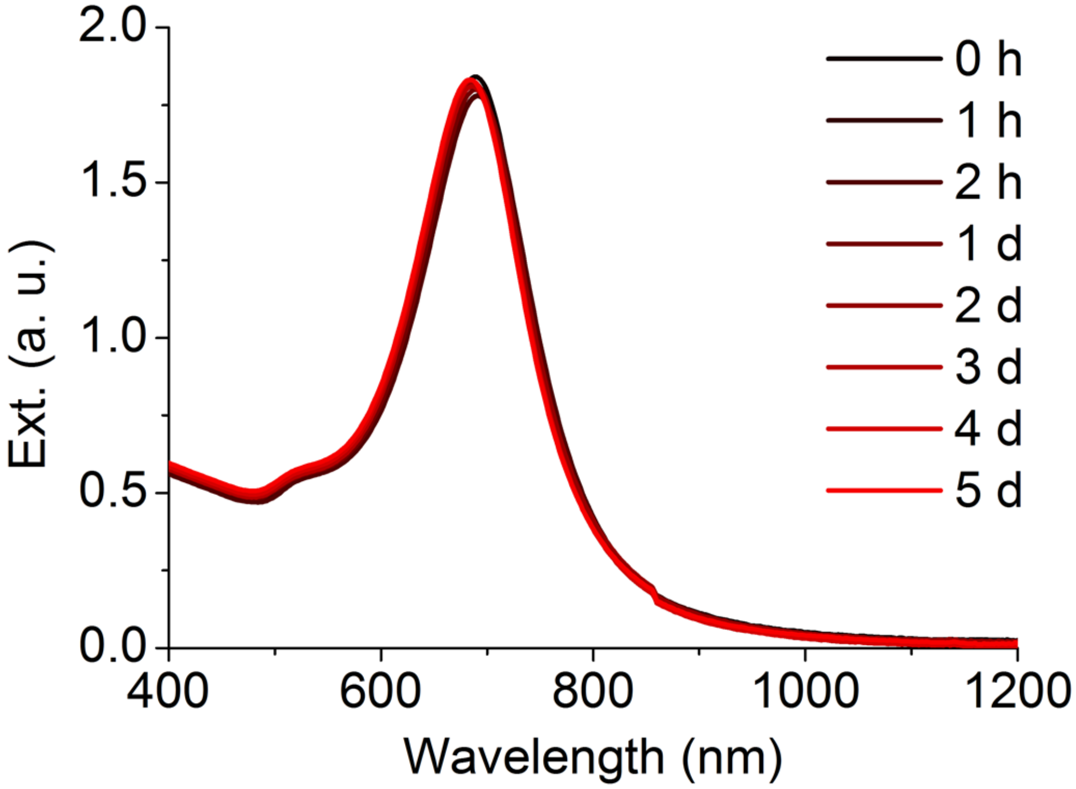

Figure S7. UV-Vis-NIR spectra of PEGylated NOPs stored at 25 °C for different aging times indicated in the legends.

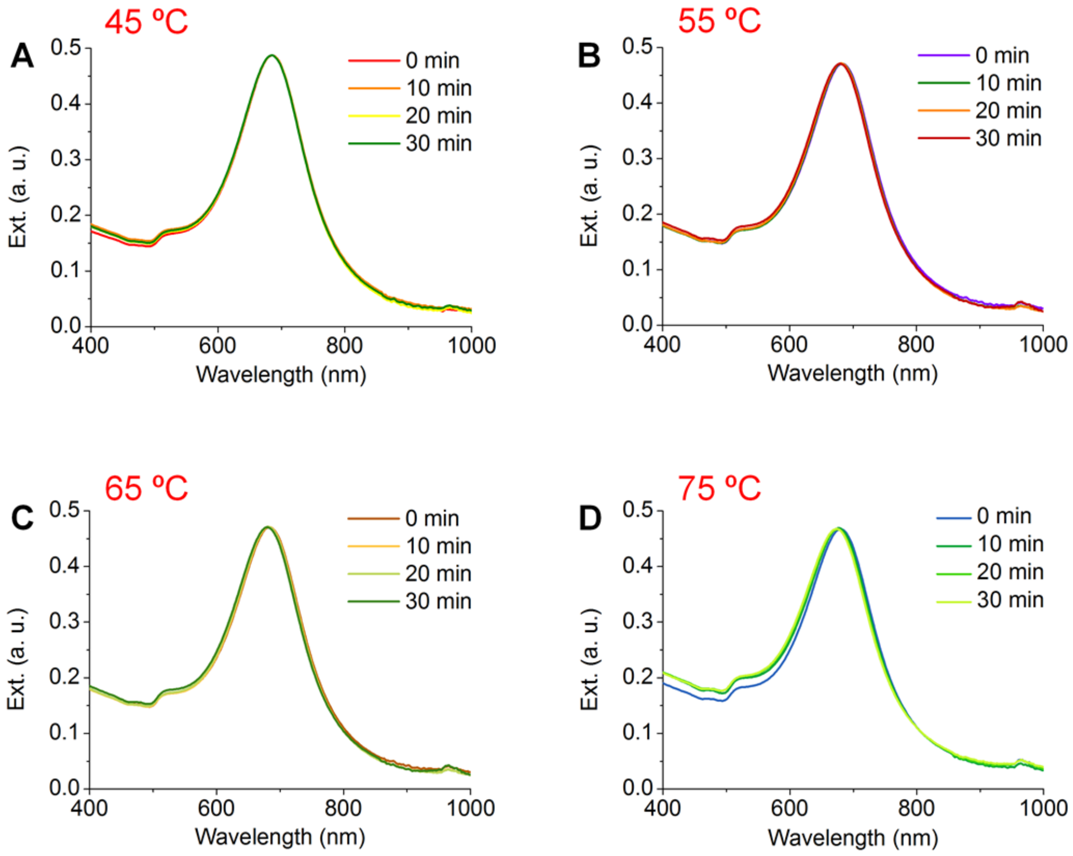

Figure S8. Thermal stability of PEGylated NOPs. UV-Vis-NIR spectra of PEGylated NOPs stored at different temperatures: (A) 45 °C, (B) 55 °C, (C) 65 °C, and (D) 75 °C. There was negligible blueshifts in the extinction spectra even at 75 °C.

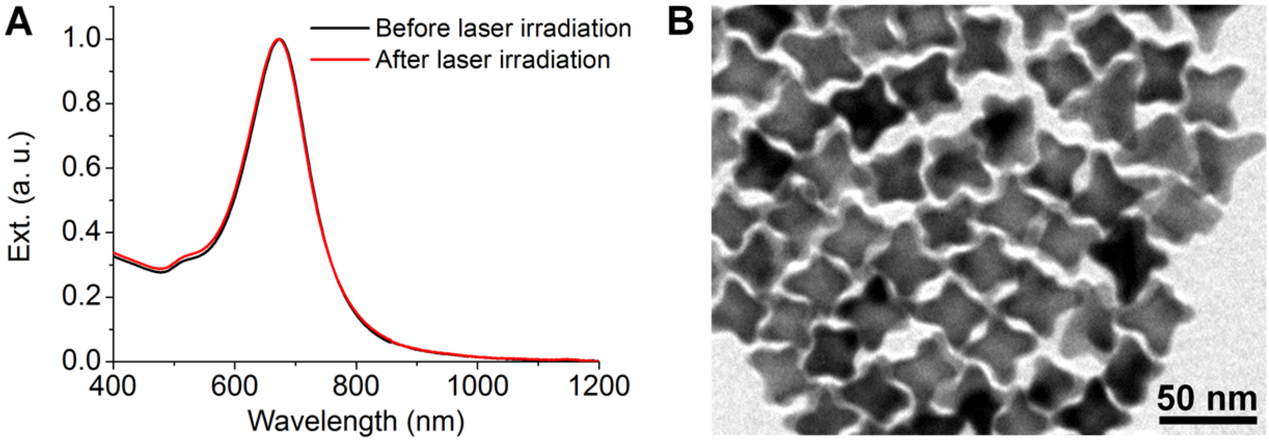

Figure S9. (A) UV-Vis-NIR spectra of PEGylated NOPs before and after the laser irradiation. (B) TEM image of PEGylated NOPs after the laser irradiation.

Table S1. Tuning the arms of NOPs by the amount of Au seeds solution.

Sample	LSPR peak (nm)	Length (nm)	Width (nm)	Aspect ratio
Seed-1.0 μL	722	22.8 ± 1.4	11.4 ± 1.0	2.00
Seed-1.5 μL	691	19.0 ± 1.3	10.2 ± 1.0	1.86
Seed-2.0 μL	666	16.4 ± 1.2	10.8 ± 1.0	1.52
Seed-3.0 μL	632	15.8 ± 1.5	12.6 ± 1.1	1.25

Table S2. Photothermal conversion efficiencies (η) of different $OD_{660\ nm}$ of NOPs irradiated at 1.0 W/cm^2 power density.

Optical density (a. u.)	Power density (W/cm^2)	η
0.10	1.0	75.8%
0.25	1.0	80.4%
0.50	1.0	83.0%
1.0	1.0	76.5%

Photothermal conversion efficiency, η, was calculated as follows:

$$\eta = \frac{hS(T_{\mathrm{max}} - T_{\mathrm{surr}}) - Q_0}{P(1 - 10^{-A})}$$

where h is the heat transfer coefficient, S is the contacting area between container and environment, T_{max} is the equilibrium temperature, T_{surr} is ambient temperature of the surroundings, Q_0 represents the heat generated by water and quartz cell under laser irradiation, P is the laser power, and A is the absorbance of NOPs at 660 nm.
